# Case for diagnosis. A pruritic eruption of keratotic papules over the face and neck^☆^^☆☆^

**DOI:** 10.1016/j.abd.2020.06.004

**Published:** 2020-11-18

**Authors:** Maria Relvas, Joana Calvão, Inês Coutinho, José Carlos Cardoso

**Affiliations:** Department of Dermatology, Coimbra University Hospital Center, Coimbra, Portugal

**Keywords:** Acitretin, Grzybowski, Keratoacanthoma

## Abstract

Generalized eruptive keratoacanthoma of Grzybowski is a rare variant of multiple keratoacanthomas counting with about 40 cases reported. It is a chronic and progressive disease for which none of the described therapeutic options has been entirely satisfactory. We report a case of an 83-year-old female who presented with a 3-month history of extremely pruritic, multiple, skin-coloured to erythematous to brownish, millimetric papules, with a keratotic centre. Histological examination of an incisional biopsy was consistent with the diagnosis of keratoacanthoma. The patient started acitretin 25 milligrams daily with a complete resolution of pruritus and regression of numerous lesions.

## Case report

**Figure 1:**
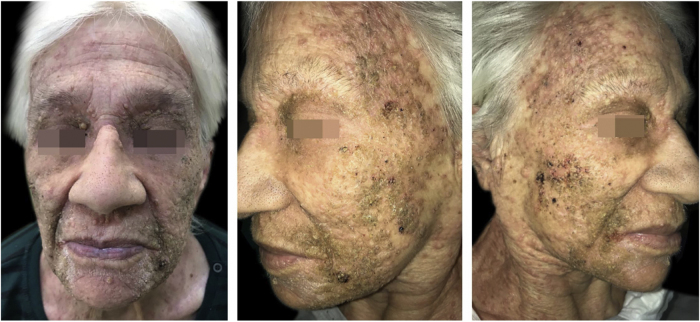
Multiple skin-colored to erythematous to brownish, 1 − 2 mm papules, with a slightly keratotic center, sometimes coalescing into verrucous plaques; koebnerization is also present.

An 83-year-old female presented with a three-month history of extremely pruritic, multiple, skin-coloured to erythematous to brownish, millimetric papules, with a keratotic center, sometimes coalescing into verrucous plaques (Fig. 1). The lesions were distributed bilaterally over the face and neck, without mucosal involvement. There was no deterioration of her general condition. She had no relevant personal or family medical history. Laboratory findings included a complete blood count and renal and liver function tests, which were all normal.

**Figure 2:**
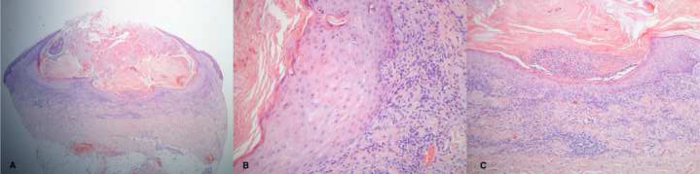
Histological examination. A, Well-demarcated, crateriform lesion filled with a predominantly orthokeratotic keratin plug, with some areas of parakeratosis. B, Surrounding epithelium showing mild irregular acanthosis and cells with abundant ground-glass cytoplasm, displaying minimal atypia and pleomorphism at the basal layer. C, Intratumoral neutrophilic microabscesses and lymphocytic inflammatory infiltrate in the underlying dermis. (Hematoxylin & eosin, A × 40; B × 200; C × 100).

Incisional cutaneous biopsy revealed a well-demarcated, crateriform lesion, whose center was filled with a predominantly orthokeratotic keratin plug, with some areas of parakeratosis (Fig. 2A). The surrounding epithelium showed mild irregular acanthosis containing abundant ground-glass cytoplasm cells, displaying minimal atypia and pleomorphism at the basal layer (Fig. 2B). Intratumoral neutrophilic microabscesses were observed, alongside with lymphocytic infiltrate in the underlying dermis (Fig. 2C). These findings were consistent with the diagnosis of keratoacanthoma.

## What is your diagnosis?

a) Muir-Torre syndrome
b) Follicular lichen planus
c) Pityriasis rubra pilaris
d) Generalized eruptive keratoacanthomas of Grzybowski

## Discussion

Correlating the clinical and histological findings, the diagnosis of generalized eruptive keratoacanthomas of Grzybowski (GEKA) was established.

GEKA is a rare variant of multiple keratoacanthomas, with only around 40 cases reported.^1^ Its onset most commonly occurs between the fifth and seventh decades, without sex predilection.^2^ Unlike other types of multiple keratoacanthomas, such as Ferguson-Smith or Witten and Zak, all cases are sporadic.^2^ It is characterized by a relatively sudden onset of hundreds to thousands of small (1−3 millimeters), skin-colored to erythematous papules with a tendency to coalesce. Some show a central umbilication containing a horny keratotic plug.^3^ They predominate on sun-exposed areas, namely the face and neck, leading to masked facies and ectropion. Furthermore, sun-protected sites, including mucous membranes, the trunk and intertriginous areas are commonly affected.^4^ The lesions tend to be severely pruritic and koebnerization is frequently reported.

The histopathological findings are similar to those observed in solitary keratoacanthomas. These comprise an exo-endophytic, well-demarcated lesion, with an invaginating keratin filled-crater, surrounded by glassy eosinophilic keratinocytes. Unlike squamous cell carcinoma, cytologic atypia is usually minimal.^5^ Sometimes the lesions may show features of proliferative or regressive stages of keratoacanthomas, so that histological diagnosis can easily be missed.

The exact etiology of GEKA is still unknown. Nonetheless, several factors have been proposed to play a role in its pathogenesis, including trauma, immunological abnormalities, ultraviolet radiation, chemical carcinogens, and viruses, particularly human papillomavirus.^1^, ^3^, ^6^

GEKA is a chronic and progressive disease for which none of the described therapeutic options has been entirely satisfactory. Surgical excision, cryotherapy, laser ablation, and radiotherapy are restricted to bigger lesions, being impractical for the remaining.^3^ Topical agents, such as 5-fluorouracil, corticosteroids, imiquimod, and tretinoin, also demonstrated little benefit.^4^

Systemic therapies, especially oral retinoids, are the preferred approach. Other reported options include methotrexate, cyclophosphamide, corticosteroids, and erlotinib, with variable results.^4^

**Figure 3:**
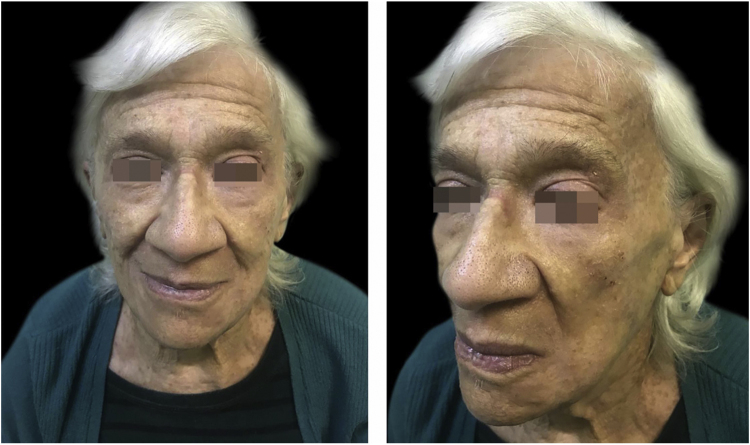
Regression of numerous lesions after 7 weeks of therapy with acitretin.

The present patient started acitretin 25 mg daily, with a complete resolution of pruritus and regression of numerous lesions (Fig. 3). The dosage was then decreased over the next five months. She is currently on 10 mg three times per week and, so far, no new lesions have emerged.

## Financial support

None declared.

## Authors' contributions

Maria Relvas: Drafting and editing of the manuscript; collection, analysis, and interpretation of data; participation in the design of the study; intellectual participation in the propaedeutic and/or therapeutic conduct of the studied cases; critical review of the literature; critical review of the manuscript.

Joana Calvão: Collection, analysis, and interpretation of data; participation in the design of the study; intellectual participation in the propaedeutic and/or therapeutic conduct of the studied cases.

Inês Coutinho: Collection, analysis, and interpretation of data; participation in the design of the study; intellectual participation in the propaedeutic and/or therapeutic conduct of the studied cases.

José Carlos Cardoso: Intellectual participation in the propaedeutic and/or therapeutic conduct of the studied cases; critical review of the literature; critical review of the manuscript.

## Conflicts of interest

None declared.
